# Histological response to injected gluteraldehyde cross-linked bovine collagen based implant in a rat model

**DOI:** 10.1186/1471-2490-6-3

**Published:** 2006-02-18

**Authors:** Murat Alkan, Beril Talim, Arbay O Ciftci, Mehmet E Şenocak, Melda Cağlar, Nebil Büyükpamukçu

**Affiliations:** 1 Department of Pediatric Surgery, Hacettepe University Medical Faculty, Sihhiye, Ankara, Turkey; 2 Department of Pediatric Pathology, Hacettepe University Medical Faculty, Sihhiye, Ankara, Turkey

## Abstract

**Background:**

The aim of present study is to investigate the short and long term histopathological alterations caused by submucosal injection of gluteraldehyde cross-linked bovine collagen based on an experimental rat model.

**Methods:**

Sixty Sprague-Dawley rats were assigned into two groups as group I and II each containing 30 rats. 0.1 ml of saline solution and 0.1 ml of gluteraldehyde cross-linked bovine collagen were injected into the submucosa of bladder of first (control) and second groups, respectively. Both group I and II were further subdivided into 3 other groups as Group IA, IB, IC and Group IIA, IIB, IIC according to the sacrification period. Group IA and IIA, IB and IIB, IC and IIC rats (10 rats for each group) were sacrificed 3, 6, and 12 months after surgical procedure, respectively. Two slides prepared from injection site of the bladder were evaluated completely for each rat by being unaware of the groups and at random by two independent senior pathologists to determine the fibroblast invasion, collagen formation, capillary ingrowth and inflammatory reaction. Additionally, randomized brain sections from each rat were also examined to detect migration of the injection material. The measurements were made using an ocular micrometer at ×10 magnification. The results were assessed using t-tests for paired and independent samples, with p < 0.05 considered to indicate significant differences; all values were presented as the mean (SD).

**Results:**

Migration to the brain was not detected in any group. Significant histopathological changes in the gluteraldehyde cross-linked bovine collagen injected groups were fibroblast invasion in 93.3%, collagen formation in 73.3%, capillary ingrowth in 46.6%, inflamatory reaction in 20%.

**Conclusion:**

We emphasize that the usage of gluteraldehyde cross-linked bovine collagen in children appears to be safe for endoscopic treatment of vesicoureteral reflux.

## Background

Endoscopic subureteric injection of various materials has become a universally accepted mode of treatment for vesicoureteral reflux in recent years. Different kinds of injection materials including polytetrafluoroethylene (Teflon), silicone, cross-linked bovine collagen and dextranomers in sodium hyaluronan (DiHA) have been used for injection with various success rates [1-4]. The long term tissue effects of these materials are still obscure due to lack of clinical and experimental data in spite of wide spectrum usage for vesicoureteral reflux and incontinence.

The aim of present study is to investigate the short and long term histopathological alterations caused by submucosal injection of gluteraldehyde cross-linked bovine collagen based on an experimental rat model.

## Methods

The experiment was approved by the institutional care and use committee, and guidelines for responsible use and human care were strictly followed during the study. Sixty adult male Sprague-Dawley rats (weight 250–270 gr.) were maintained in temperature-controlled cages and a dark-light cycle, with free access to water and food. Surgical procedures were performed under general anaesthesia induced with an intraperitoneal instillation of pentobarbital. The bladder was exposed through identically opened and closed midline vertical abdominal incisions using a sterile technique.

The rats were divided randomly into two groups (I and II) each containing 30 rats. Group I (control group); 0.1 ml of saline solution was injected at the dome of bladder submucosa via a PPD injector needle. The same procedure was carried out with 0.1 ml of gluteraldehyde cross-linked bovine collagen in the other group (Group II). We did not cut or put a mark suture at the dome of the bladder.

Both group I and II were further subdivided into 3 other groups as Group IA, IB, IC and Group IIA, IIB, IIC according to the sacrification period. Group IA and IIA, IB and IIB, IC and IIC rats (10 rats for each group) were sacrificed 3, 6, and 12 months after surgical procedure, respectively.

Bladder and brain of all animals were taken for histopathological evaluation after sacrification. The materials were fixed in formaldehyde, sections were taken from the injection site, embedded in paraffin blocks and cut and stained by haematoxylin and eosin (H & E) and trichrome stains. Two slides prepared from injection site of the bladder were evaluated completely for each rat. The slides were examined while unaware of the groups and at random by two independent senior pathologists to determine the fibroblast invasion, collagen formation, capillary growth and inflammatory reaction. Additionally, randomized brain sections from each rat were also examined to detect migration of the injection material. The measurements were made using an ocular micrometer at ×10 magnification. The results were assessed using t-tests for paired and independent samples, with p < 0.05 considered to indicate significant differences; all values were presented as the mean (SD).

## Results

**Figure 1:**
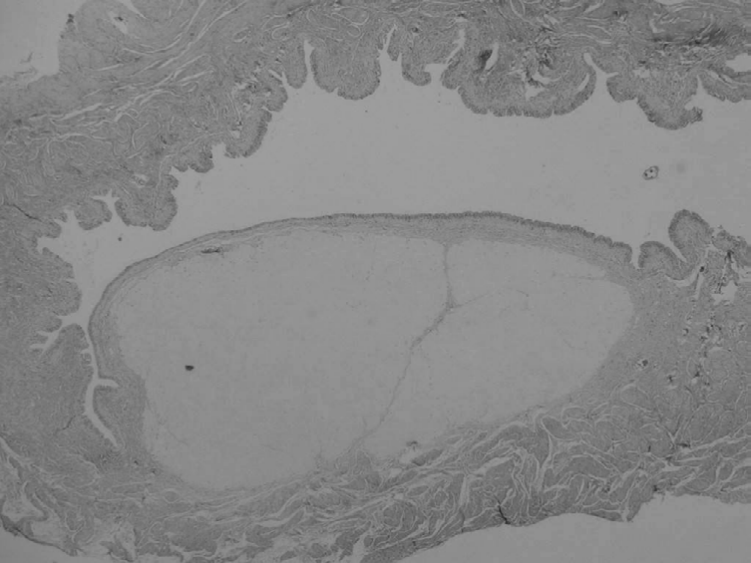
Histological section 12 months after injection of bovine collagen. The implant material persists as a mass narrowing the lumen. (H & E, original magnification ×4).

**Figure 2:**
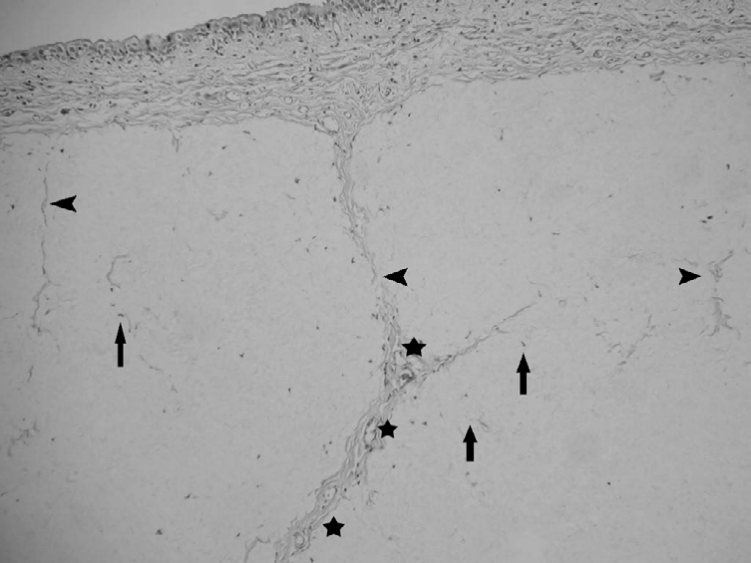
Histological section 6 months after injection of bovine collagen. Note the presence of invasion by fibroblasts (arrows), collagen formation (head arrows) and capillary growth (). (H & E, original magnification ×20).

**Figure 3:**
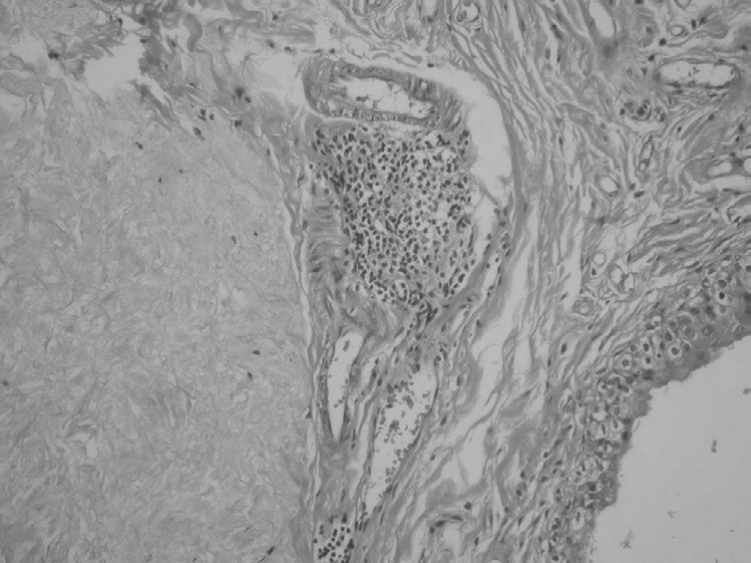
Histological section 6 months after injection of bovine collagen, showing focal lymphocytic infiltration around the implant material. (H & E, original magnification ×40).

There was no pathological finding in Group IA, IB, IC. Gluteraldehyde cross-linked bovine collagen was observed as intact and surrounded by a fibrous capsule (Figure 1). Microscopical evaluation revealed that major histopathological reactions to the injected material were fibroblast invasion 93.3%. Collagen formation surrounding the material was in 73.3%. Capillary ingrowth, especially which was more severe in the first and second three months samples 46.6%. Minimal inflammatory reaction was a prominent finding observed in the 6 months samples (Group IIB) 20% (Figure 2, figure 3). Eosinophilic infiltration, foreign body type giant cells, granuloma formation, calcification or any other pathological reactions were not observed in either bladder or brain samples. Migration was also not observed in the brain samples (Table 1).

## Discussion

Endoscopic subureteric technique corrects reflux in children with good results in many series [1-4]. Various implant materials have been used so far and the long term efficacy of implant materials still remains controversial. The best injectable material should be easy to inject, without systemic side effects, and should make a good augmentation in the subureteral plane of the ureter orifice. The material should not induce granuloma formation or migrate to the distant organs. It should not incite an allergic or inflammatory response.

The subureteric injection of polytetrafluoroethylene (Teflon) was first utilized in 1981 by Matouschek [5]. This technique has been developed and used by O' Donnell and Puri who reported their results in both pigs and humans with experimental and clinical studies in 1984 [6]. At first, polytetrafluoroethylene (Teflon paste) was used for subureteric injection and called subureteric polytetrafluoroethylene injection (STING).

Complications of various injection materials (migration, granuloma formation, allergic reactions and systemic side effects) have forced the investigators to find out the best injection material. In 1983 Mittlemann et al. reported pulmonary Teflon granuloma after periurethral injection for urinary incontinence [7]. Experimental studies of Malizia et al. in 1984 demonstrated that Teflon particles migrated to pelvic lymph nodes, lungs, brain, kidney and spleen of monkeys 10.5 months after periurethral injections. At 10.5 months, they found Teflon granulomas at injection sites and distant migration [8]. In 1993 Aaronson et al.'s experimental study has shown that Teflon particles migrate not only to the lungs but also pass through the pulmonary vascular bed to migrate to the brain [9]. Then the polydimethylsiloxane (Macroplastique) paste has been used as an alternative material. Macroplastique in the canine model revealed migration of the particle to the capsule of spleen (Preston Smith in 1994) [10]. Travis et al. reported a case of silicone-induced granuloma formation in an inguinal lymph node nine years after silicone rubber had been used in hip arthroplasty in 1984 [11]. In spite of confirmed granuloma formation following Teflon and silicone injections, no proof of malignancy has been shown in the human after long term usage [9-11].

In the recent years bovine collagen and dextranomers in sodium hylauronan (DiHA) have been used more frequently for the endoscopic treatment of vesicoureteral reflux [2,4,12,13].

Dextranomers in sodium hyaluronan consists of dextranomer microspheres of 80–120 μm. diameter in a 1% molecular weight of NaHA solution. 1 ml volume contains 0.5 ml dextranomer microspheres and consists of a network of cross-linked co-polymers of dextran [14].

Stenberg et al. have done clinical and experimental studies with DiHA revealing that the material was stable and giant cells between microspheres, ingrowth of fibroblasts, collagen around dextranomers and granulomatous inflammatory reaction of the foreign body type without tissue necrosis and calcification were the histopathological findings [15,16]. The chronic inflammatory response which is stimulated with the presence of the inert material, becomes grossly visible and circumscribed pale mass which is called granuloma or granuloma formation. Granulomatous reaction is the same situation of which grossly visible and circumscribed pale mass has not occurred yet. They also questioned the possible malignant transformation of granulomas after Teflon or silicone injections based on the studies of Dewan [17] and Hatanaka [18].

Even DiHA seems to be a good injection material with collagen formation surrounding the spheres, there was a decrease in implant volume by 23% over a period of 12 months [15]. With the following years, decrease in the implant volume may be significant because of the small diameter of spherical masses in the implant. We do not know the importance of the variation in the diameter of the spherical masses and the hypertrophy of the lining cells in some of the spheres.

Bovine collagen is composed of highly purified bovine dermal collagen (95% type I and 5% type III) that is cross-linked with gluteraldehyde and dispersed in phosphate-buffered physiological saline [19]. The histological behaviour of gluteraldehyde cross-linked bovine collagen implants in the bladders of experimental animals and in humans has been examined and invasion of host fibroblasts into the bovine implants *without *causing granuloma formation and collagen formation has been detected [20,21].

In the light of our findings and literature information, the histological behaviour of bovine collagen implants in the bladder of experimental animals and the implants removed at open surgery showed nearly the same histological properties [20,22-24].

The collagen deposit is typically well encapsulated by collagen, created a mass effect with the surrounding tissue and persisted at least up to12 months, causing minimal inflammatory reaction. Inflammatory reaction was detected only at 6 months. Because of minimally inflammatory reaction in the 3 and 12 months groups, inflammatory reaction was not observed. Collagen deposit surrounded by fibrous capsule may result for a- long time persistence of the material in the injected site. On the other hand DiHA spheres were detected in various small diameters. We do not know which one is better. Addition to these, minimal inflammatory reaction may be the advantage of the usage of collagen to the other materials.

In this study, we also could not detect eosinophilic infiltration, foreign body type giant cells, granuloma formation and calcification.

We did not investigate the lungs, liver and pelvic lymph nodes. Only bladder and brain tissue were examined. Our purpose was to examine the histopathological changes at the injection site in the bladder. Sure, it would be better to inject this material to the trigon of the bladder but it is difficult to find out and inject into the trigon of the rat bladder. In this study brain tissue was examined and migration was not detected. But brain sections were not examined under electron microscope as Aaronson examined the migration before [9]. Of course our results still does not completely exclude the possibility of migration.

## Conclusion

It should be kept in the mind that long-term effect(s) of this process is still unknown. For this reason, future research is needed to find out long-term effects of these injection materials.

We emphasize that the usage of gluteraldehyde cross-linked bovine collagen in children seems to be safe but further prospective human (and/or animal) studies are required to clarify the long term effects with regard to clinical applications.

## Abbreviations

DiHA: dextranomers in sodium hyaluronan.

## Competing interests

The author(s) declare that they have no competing interests.

## Authors' contributions

MA: study design, experiments, and drafting of the manuscript. BT: histopathological study. AOC: study design and performed the statistical analysis. MES: study design. MC: histopathological study. NB: study conception, study design and coordination. All authors have read and approved the final manuscript.

## Pre-publication history

The pre-publication history for this paper can be accessed here:



## Figures and Tables

**Table 1:** Histopathological Findings

Gluteraldehyde cross-linked bovine collagen (Injection material)	Group IIA 3 months later (10 rats)	Group IIB 6 months later (10 rats)	Group IIC 12 months later (10 rats)	Total (30 rats)
Fibroblasts invasion	10 (100%)	8 (80%)	10 (100%)	28 (93.3%)
Collagen formation	8 (80%)	6 (60%)	8 (80%)	22 (73.3%)
Capillary growth	6 (60%)	6 (60%)	2 (20%)	14 (46.6%)
Inflammatory reaction	0 (0%)	6 (60%)	0 (0%)	6 (20%)
Foreign body type giant cells	0 (0%)	0 (0%)	0 (0%)	0 (0%)
Granuloma formation	0 (0%)	0 (0%)	0 (0%)	0 (0%)
Calcification	0 (0%)	0 (0%)	0 (0%)	0 (0%)
Migration to brain	0 (0%)	0 (0%)	0 (0%)	0 (0%)
